# Analysis of Maternal Coronavirus Infections and Neonates Born to Mothers with 2019-nCoV; a Systematic Review

**Published:** 2020-04-15

**Authors:** Salut Muhidin, Zahra Behboodi Moghadam, Maryam Vizheh

**Affiliations:** 1 Department of Management, Macquarie Business School, Macquarie University, New South Wales, 2109, Australia.; 2 Department of Reproductive Health and Midwifery, School of Nursing and Midwifery, Tehran University of Medical Sciences, Tehran, Iran.

**Keywords:** COVID-19, severe acute respiratory syndrome coronavirus 2 (SARS-CoV-2), pregnancy outcome, infectious disease transmission, vertical, infant, newborn, systematic review

## Abstract

**Introduction:**

The emergence and fast spread of 2019 novel coronavirus (2019-nCoV) threatens the world as a new public health crisis. This study aimed to clarify the impact of novel coronavirus disease (COVID-19) on pregnant patients and maternal and neonatal outcomes.

**Methods:**

A comprehensive literature search was conducted in databases including PubMed, Scopus, Embase, ProQuest, and Science Direct. All studies including original data; case reports, case series, descriptive and observational studies, and randomized controlled trials were searched from December 2019 until 19 March 2020.

**Results:**

The search identified 1472 results and 939 abstracts were screened. 928 articles were excluded because studies did not include pregnant women. Full texts of eleven relevant studies were reviewed and finally nine studies were included in this study. The characteristics of 89 pregnant women and their neonates were studied. Results revealed that low-grade fever and cough were the principal symptoms in all patients. The main reported laboratory findings were lymphopenia, elevated C-Reactive Protein (CRP), Amino alanine transferase (ALT), and Aspartate amino transferase (AST). In all symptomatic cases, chest Computerized Tomography (CT) scans were abnormal. Fetal distress, premature rupture of membranes and preterm labor were the main prenatal complications. Two women needed intensive care unit admission and mechanical ventilation, one of whom developed multi-organ dysfunction and was on Extracorporeal Membrane Oxygenation (ECMO). No case of maternal death was reported up to the time the studies were published. 79 mothers delivered their babies by cesarean section and five women had a vaginal delivery. No fetal infection through intrauterine vertical transmission was reported.

**Conclusions:**

Available data showed that pregnant patients in late pregnancy had clinical manifestations similar to non-pregnant adults. It appears that the risk of fetal distress, preterm delivery and prelabor rupture of membranes (PROM) rises with the onset of COVID-19 in the third trimester of pregnancy. There is also no evidence of intrauterine and transplacental transmission of COVID-19 to the fetus in the third trimester of pregnancies.

## Introduction

The emergence and fast spread of 2019 novel coronavirus (2019-nCoV) threatens the world as a new public health crisis (1). Severe Acute Respiratory Syndrome Coronavirus 2 (SARS-CoV-2) is a member of the Coronavirus family, whose members cause a range of infectious diseases from a common cold to Severe Acute Respiratory Syndrome (SARS) and the Middle East Respiratory Syndrome (MERS) (2). At the moment, there is very limited knowledge about the various aspects of the disease including pregnancy and maternal and fetus health. Epidemic and pandemic viral infections in recent years have shown that pregnant women experience more adverse outcomes than non-pregnant ones (2). The risk of developing a viral infection, including SARS-CoV, MERS-CoV, Ebola, H1N1, and influenza-A is higher in pregnant women, and these infections can lead to undesirable medical and prenatal outcomes such as maternal mortality, spontaneous abortion, stillbirth and preterm delivery. However, we know little about COVID-19 in pregnancy (3). 

Pregnancy-related immunological and physiological changes that naturally occur during pregnancy can lead to worsening of respiratory infections due to systemic effects on the body. Increased heart rate, oxygen consumption, stroke volume, and decreased pulmonary capacity and functional residual capacity are the main physiological changes in the cardiovascular and respiratory systems during pregnancy that increase the complications of COVID-19 in pregnant women compared to the non-pregnant population (2, 4). On the other hand, pregnancy is associated with immunosuppression. This situation makes pregnant women more susceptible to infectious diseases (2). Moreover, there is a possibility of vertical transmission of SARS-CoV-2 from mother to fetus and creating significant infections in fetuses and neonates (2). These concerns are based on experiences gained from infections such as the Zika virus, Ebola virus, Marburg virus and other agents capable of transmitting vertically from mother to fetus. These viruses could threaten the health and survival of the infected mother and her fetus (4).

Currently, the clinical attributes and vertical transmission capability of COVID-19 in pregnant patients are obscure. This raises questions about COVID-19 in pregnancy. These questions include: Are the symptoms of COVID-19 infection different in pregnant women compared to the general population? Are pregnant women with COVID-19 pneumonia at a higher risk of death? Are pregnant women with COVID-19 pneumonia at higher risk of obstetrics complications such as preterm labor and low birth weight? Can infection transmit vertically and cause fetal and neonatal disease? Therefore, it is crucial to find answers to these questions to gain insight and design a management protocol for pregnant women. So, this study was designed to investigate the impact of COVID-19 on pregnant women and their infants. Moreover, assessing the risk of vertical transmission and the possible mechanisms of infection transmission from mother to fetus were other aims of this review.

## Methods

***Study design*** ***and*** ***Search strategy***

This study is a systematic review conducted to identify the research studies that addressed the impact of COVID-19 in pregnancy. 

**Figure 1:**
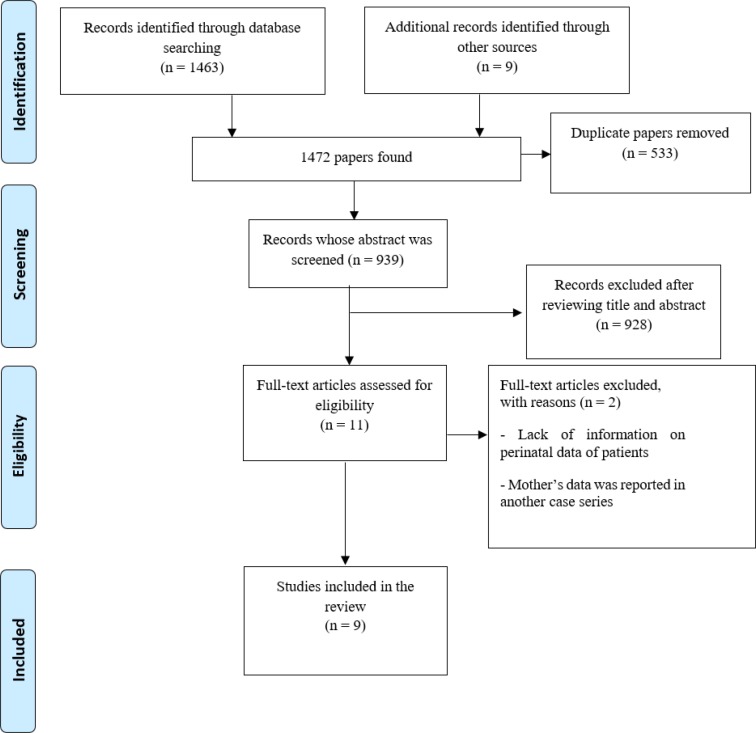
Flow chart of the study’s selection process based on PRISMA

Since, this emerging pneumonia was first reported from China in December 2019, a comprehensive literature search was conducted in databases from its inception in December 2019 until 19 March 2020. All studies including original data; case reports, case series, descriptive, observational and randomized controlled trials were included. Searching articles was carried out using related keywords in several international electronic databases, namely: PubMed, Scopus, Embase, ProQuest, and Science Direct. Additionally, a manual search was performed in Google Scholar. References of the included studies were reviewed manually. The article search was performed using the keywords: “covid-19 OR SARS-CoV-2 OR novel coronavirus AND pregnancy OR prenatal OR neonatal OR fetus”. Some of search strategies were: "COVID-19"[All Fields] OR "severe acute respiratory syndrome coronavirus 2"[Supplementary Concept] OR "severe acute respiratory syndrome coronavirus 2"[All Fields] OR "2019-nCoV"[All Fields] OR "SARS-CoV-2"[All Fields] OR "2019nCoV"[All Fields] OR (("Wuhan"[All Fields] AND ("coronavirus"[MeSH Terms] OR "coronavirus"[All Fields])) AND ("pregnancy"[MeSH Terms] OR "neonatal"[All Fields]). The selection process of the studies is represented in Fig. 1. 

***Eligibility criteria***

Inclusion criteria were: articles published in English or Persian language that contained the keywords in their title, abstract or keywords, and their full-text was available. Articles that addressed neonates without providing a detailed perinatal history of their mothers, articles of pregnant patients without reporting perinatal data and outcomes and articles in other languages were excluded. Moreover, to avoid overlapping in data, systematic reviews were excluded. 

***Study selection and data extraction***

Primary research articles that assessed various aspects of COVID-19 in pregnancy were included in this study. The initial database searching and screening the titles and abstracts were performed by one author (MV). Then these articles were classified as: relevant, irrelevant or unsure. All authors (SM, ZB, and MV) assessed every single article classifying them as relevant or unsure, independently. Once all relevant articles were identified, all authors reviewed full texts of all identified articles and extracted the data. After completing primary data extraction, all authors assessed the content of retrieved data. 

The following data were obtained from each included study: characteristics of the study including first author's name, setting and date of publication, eligibility criteria, and sample size; details on patients including demographic, epidemiological, clinical and para-clinical manifestations, prenatal complications, maternal and fetal outcomes, and adverse effects related to COVID-19, were extracted from the included articles using a pre-defined data extraction checklist, developed by authors. Once data extraction was complete, the authors reviewed the obtained data and summarized them. 

***Methodological quality (risk of bias) assessment***

Quality assessment tools of the National Institutes of Health (NIH) consisting of 9 items for case-report studies and 12 items for case-control studies were used to assess the quality and risk of bias of included studies by two authors (ZB, MV), independently (5, 6) (Table 3).

***Outcome of interest***

The clinical manifestations of pregnant mothers with COVID-19, the risk of death, the possibility of vertical transition of infection and obstetrics and neonatal outcomes of COVID-19 were the studied outcomes in this systematic review. 

**Statistical analysis**

SPSS version 16 was used for analyzing data. Categorical variables are expressed as number (%) and continuous variables are expressed as ranges.

## Results

The search yielded 1472 results and 939 abstracts were screened. 928 articles were excluded because the studies did not include pregnant women. Full texts of eleven relevant studies were reviewed. After assessing full-texts, two studies were excluded. One of them was excluded because the mother’s data was reported in another case series. Another study that reported “clinical, and CT imaging features of COVID-19 pneumonia” in seventeen pregnant mothers was also excluded due to lack of information on obstetrics and perinatal data of patients (Figure 1). All nine included studies that reviewed pregnant women and their infants are summarized in Table 1. 

**Table1:** Characteristics, objectives and outcomes of included articles

Row	Author[Ref] Setting Date of publication	Design	Objective (s)	Main outcome(s)
1	Chen et al. (7) China Feb 12, 2020	Retrospective case series	A) assessing the clinical attributes of COVID-19 in pregnancy B) Assessing the risk of vertical transmission of COVID-19 infection	A) COVID-19 pneumonia in pregnant women showed attributes similar to non-pregnant adult patients B) There is yet no evidence for intrauterine vertical transmission of COVID-19 from mother to fetus
2	Zhu et al. (8) China Feb 10, 2020	Retrospective case series	Assessing clinical manifestation of 10 neonates born to mothers with COVID-19 pneumonia	A) There is a possibility of adverse effects of perinatal COVID-19 infection on newborns such as premature labor, fetal distress, respiratory distress, thrombocytopenia due to abnormal liver function, and death B) Vertical transmission of COVID-19 is not currently reported.
3	Li et al. (9) China 2020	Case-control study	Assessing maternal and neonatal outcomes of pregnant women with COVID-19 pneumonia	A) There was no evidence that COVID-19 pneumonia is accompanied with severe maternal and neonatal complications B) Considering the delay in receiving PCR tests, in the third trimester of pregnancy, especially in epidemic areas, chest CT scans can be an effective way of screening for COVID-19 pneumonia
4	Liu et al. (10) China Feb 2020	Case series	Clinical symptoms and outcomes of COVID-19 infection in pregnant patients	COVID-19 infection can pose a threat to maternal and neonate’s health
5	Fan et al. (2) China 2020	Case report	Assessing perinatal transmission of COVID-19	A) Perinatal outcomes were uneventful B) The risk of vertical transmission of COVID-19 is trivial
6	Chen et al. (11) China 16 Mar 2020	Case series	Assessing the safety of general or epidural anesthesia in pregnant patients with COVID-19	A) Both general and epidural anesthesia could be applied safely in pregnant patients with COVID-19 undergoing cesarean section B) There was a higher rate of hypotension during epidural anesthesia
7	Wang (3) China 2020	Case report	Explaining a case of preterm delivery in a pregnant woman with Coronavirus	Neonate was unaffected by COVID-19
8	Liu et al. (12) China 25 Feb2020	Case series	Assessing the outcomes of COVID-19 during pregnancy	A) Perinatal outcome in both mothers and infants was uneventful B) There was no evidence of vertical transmission
				
9	Song et al. (13) China, 2020	Case report	Explaining anesthetic managementof a parturient with COVID-19	Applying both epidural and spinal anesthesia provided effective anesthesia.

Overall, in these nine studies, 89 pregnant women were studied, 68 of which were confirmed to have COVID-19 pneumonia through positive result of rt-PCR for SARS-CoV-2 and eighteen were suspected cases admitted to hospital for their symptoms or labor. Three mothers were discharged with an uncomplicated ongoing pregnancy. Moreover, the characteristics of 89 neonates born to 86 infected mothers singletons and three sets of twins) were studied. The overall risk of bias in the included studies was low (Table 2). Two of the seven case-report studies failed to adequately follow up patients (11, 12). In all studies except for 2, the statistical method was either not reported or did not need to be reported. In the case-control study, the process of choosing sample size and adjustment of confounding variables was not clear (9). To more focus on the objectives and topics discussed in the articles, the results of this review will be presented in several sections.

**Table 2:** Quality assessment of included studies

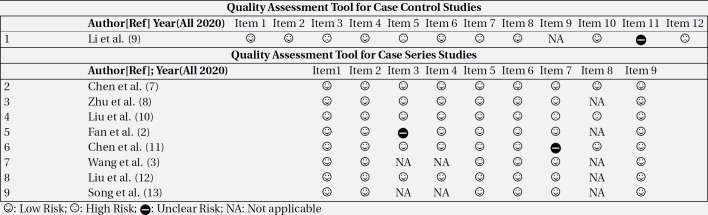

***Clinical features, Diagnosis, and Treatment:***

Based on data retrieved from relevant articles, pregnant patients ranged from 22 to 40 years old. All women were in the third trimester of pregnancy. Fever and cough were the dominant and principal symptoms in all patients. But in none of them did the temperature rise above 39 degrees. Fever was low-grade in all patients. Fatigue, dyspnea, and sore throat were less common symptoms. Also, three women reported gastrointestinal symptoms such as diarrhea or vomiting. 

Between 18 to 100 percent of the patients in any individual study and overall, 40 patients had a clear history of epidemiological exposure to COVID-19, either exposure to the relevant environment or contact with an infected person. One study did not provide a history of how patients were infected (9).The time interval between onset of symptoms to delivery varied from 15 days before to 3 days after delivery.

Diagnosis of COVID-19 in the mentioned studies followed the standard protocol, like non-pregnant patients. For this purpose, clinical symptoms, history of exposure to infection, laboratory results, computed tomography (CT) evidence of pneumonia and typical signs of viral infection were considered and eventually all cases were confirmed via quantitative RT-PCR (real-time reverse transcription-polymerase chain reaction) on SARS-CoV-2. In all studies, maternal throat swab samples were collected and tested for SARS-CoV-2 with the Kit (BioGerm, Shanghai, China) recommended by Chinese Center for Disease Control and Prevention (CDC) following WHO guidelines for qRT-PCR (8). 

Laboratory findings such as lymphopenia (N=19), elevated C-reactive protein (N=29); alanine aminotransferase (ALT) and aspartate aminotransferase (AST) (N=3) and decreased Albumin (N=1) were reported in most studies. 

In all symptomatic cases, chest CT scans revealed abnormal viral changes in lungs demonstrating lungs with patchy ground-glass shadows.

Oxygen therapy, antiviral and antibiotic therapy was prescribed as standard treatment based on each patient’s condition. Additionally, two patients were admitted to intensive care unit (ICU), and one patient was on extracorporeal membrane oxygenation (ECMO). 

***Obstetric characteristics and outcomes:***

Previous medical history of patients revealed that 30 mothers had a history of other comorbidities before the infection with COVID-19 including hypertension (HTN) (n=5) (7, 9, 11)**, **pre-eclampsia (n=2) (7, 9), inﬂuenza (n=1) (7), vaginal bleeding in the third trimester (n=2) (2, 8), hypothyroidism (n=3) (9, 12), polycystic ovary syndrome (n=3) (9), hepatitis B (n=3) (9), gestational diabetes mellitus (n=6) (9, 11, 12) and anemia (n=5) (11). In the study by Li et al., around 70% had other maternal complications, which was significantly higher than the control group (31-33%). All these complications were developed before diagnosis of pneumonia (9).

Moreover, fetal distress (N=15) (7-10, 12), prelabor rupture of membranes (PROM) (N=6) (7-9), Preterm labor (N=30) (2, 3, 7-11, 13), abnormal umbilical cord (N=2) (8), stillbirth (N=1) (10), placenta previa (N=1) (8), abnormal AF (N=2) (8), placental abruption (N=1) (9) were the most commonly reported complications. One study with 17 samples did not provide complete details on maternal or neonatal complications (11).

In the study by Chen et al., six of the nine studied women had postpartum fever (7). Furthermore, in the study by Li et al., eight of the 16 patients with confirmed COVID-19 pneumonia and six of the eighteen suspected cases reported postpartum fever, 50.0%, and 33.3%, respectively(9). Two women were admitted to the ICU and required mechanical ventilation, one of whom developed multi-organ dysfunction. Up to the time of publishing the study, the mentioned patient was on extracorporeal membrane oxygenation (ECMO) (3, 10). No cases of maternal death were reported up to the time the studies were published. 

***Mode of delivery:***

Fear and uncertainty about the risk of vertical transmission of infection during vaginal delivery, have made confirmed or suspected COVID-19 pneumonia an indication for cesarean section in china since 24 January 2020(5, 9). 

In the reviewed papers, 79 of 86 mothers delivered their babies by cesarean section. Although the main indication of cesarean section was COVID-19, fetal distress (N=11) (7, 8, 10), pre-eclampsia (N=1) (7), a history of C-section (N=2) (7, 13), premature rupture of the membrane (N=7) (7, 8, 10), stillbirth and a history of stillbirth (N=2) (10), abnormal amniotic fluid (N=2) (8), abnormal umbilical cord (N=2) (8), abnormal placenta (N=1) (8), persistent fever (N=1) (2), and admission to the intensive care unit (ICU) (N=1) (3), were additional indications that led to caesarean section, especially emergency cesarean section. Five of the patients had natural vaginal delivery (8, 9, 12). Two of them had natural vaginal delivery (NVD) because during admission for full-term labor they did not show any respiratory symptoms. One of them developed fever two days after childbirth and in another patient, on the same day of labor CT images of right lung changed to the patchy shadows (9). Moreover, one normal vaginal delivery was performed due to maternal request (12). 

***Vertical transmission potential of COVID-19 infection***

In a study conducted by Chen et al. for assessing the possibility of vertical transmission, they examined amniotic ﬂuid, umbilical cord blood, breast milk samples and neonatal throat swab in nine pregnant women with COVID-19 pneumonia and they concluded that there is no evidence of vertical transmission of SARS-CoV-2 infection (7). Moreover, Liu et al. carried out further assessments by examining placenta tissue and vaginal mucus and did not report any infected neonates (10). Also, the findings of studies carried out by Fan et al. and Song et al. are consistent with these results (2, 13). Although in other studies, researchers did not evaluate the products of conception, by evaluating throat swabs of neonates born to these infected mothers, they reported that in all neonates, qRT-PCR was negative. Therefore, they claimed that there was no evidence of intrauterine transmission of infection. To date, none of the studies has reported neonatal infection acquired from pregnant women with COVID-19 pneumonia.

***Neonates’ outcomes:***

Data of 89 neonates was included in this review. The range of fetal weight was 1520–3820 grams. Seven infants were born with low birth weight. Also, two infants were small-for-gestational-age (SGA), and one was large-for-gestational-age (LGA) (8). None of the neonates received antiviral treatment. The results of qRT-PCR proved that none of the neonates were infected with COVID-19. Death of two neonates was reported (8, 12). 

There is a wide range of neonatal symptoms reported in these studies. Zhu et al. examined 10 neonates born to mothers with COVID-19 pneumonia comprehensively, nine of whom showed symptoms after birth. Shortness of breath in 6 cases, fever in 2, and tachycardia in one neonate were reported (8). Furthermore, four of ten neonates in one study showed a range of gastrointestinal symptoms such as gastric bleeding, feeding intolerance, refusing milk and bloating (8). Seven neonates had abnormalities in chest radiography at the time of admission, which were caused by infections (N=4), neonatal respiratory distress syndrome (NRDS) (N=2), and pneumothorax (N=1). Refractory shock was found in one of the neonates, which developed into multiple organ failure and disseminated intravascular coagulation (DIC). As a result, treatment started with platelet transfusion, suspended red blood cells, and plasma. However, he died nine days after birth. Another neonate received intravenous transfusion of gamma globulin, platelets, suspended red blood cells, and plasma. Up to the date of publishing the report, this infant was alive (8). Both of the two neonates reported by Fan et al. (2), showed complications after birth. One of them had lymphopenia, abdominal distension and low-grade fever three days after birth and diffuse haziness in chest radiograph four days after birth. However her response to the antibiotic was good and she was later discharged. The other neonate had lymphopenia (10.5%) and mild neonatal pneumonia and was treated with antibiotics and was also discharged from the hospital (2). Moreover, a 40-week baby developed Meconium Stained Amniotic Fluid and chorioamnionitis as a result of chronic fetal distress (12). Other neonates did not show any adverse outcomes. Additionally, breastfeeding was prohibited in all cases, and infants were fed with formula.

## Discussion

Data retrieved from included studies revealed that pregnant women with COVID-19 showed symptoms similar to those reported in non-pregnant adults. In the current study, the most dominant initial symptoms were fever and cough. However, there wasn’t any high-grade fever. In all patients complaining of fever, the temperature was less than 39 °C. This is consistent with the results of non-pregnant patients. One meta-analysis reviewed data of 46248 infected patients with COVID-19 and reported that in the general population, the principal clinical symptoms were fever (91±3, 95%CI 86-97%), cough (67±7, 95% CI 59-76%), fatigue (51±0, 95% CI 34-68%) and dyspnea (30±4, 95% CI 21-40%) (14). Similarly, previous studies focusing on SARS and MERS revealed that laboratory and clinical findings in pregnancy were similar to those reported in the general population (15). This study also found that the rate of developing severe pneumonia and the need for admission to ICU were relatively low and similar to the non-pregnant cases. Only two of the 86 pregnant women in this study developed severe pneumonia. However, there was not any report of maternal death. The limited data available on the effects of SARS and MERS in pregnancy, proposes that the complications of COVID-19 in pregnancy are lower than other coronaviruses. A review on SARS in 12 pregnancies revealed a case-fatality rate of 25%. Acute respiratory distress syndrome (ARDS) (N=4), disseminated intravascular coagulopathy (N=3), renal failure (N=3), secondary bacterial pneumonia (N=2), and sepsis (N=2) were the main complications (15). In pregnant women infected with SARS, mechanical ventilation was three times more common than non-pregnant cases. On the other hand, in a study on 13 pregnant cases with MERS-CoV, three patients (23%) died (15).

The standard protocol for diagnosis of COVID-19 is using real-time polymerase chain reaction (RT-PCR). Samples can be obtained from the upper respiratory tract (nasopharyngeal and oropharyngeal swabs, saliva) or the lower respiratory tract (sputum, endotracheal aspirate or bronchoalveolar lavage) as well as urine and stool. If required, samples should be repeated to confirm the diagnosis. COVID-19 can be ruled out if two consecutive samples from the respiratory tract with at least 24 hours interval are negative. Using serology as a diagnostic procedure is recommended only when RT-PCR is not available (16). However, Li et al. suggested that chest CT scans in the third trimester in pregnant women could be an effective test to assess COVID-19 pneumonia given the strained time and availability of PCR tests, especially in regions struggling with ongoing epidemics (9).

Data reported from other similar viral infections such as SARS, MERS, and influenza in previous studies showed that these infections could compromise pregnancies. The rate of poor fetal outcomes, abortion, and miscarriage was significant. Also, the outcomes of pregnant patients were more undesirable than the general population (2). In a study, 10 out of 11 pregnant mothers infected with SARS-CoV, revealed adverse outcomes. Their neonates also showed complications such as admission to neonatal ICU (NICU) (N=6, 55%) and death (N=3, 27%). Moreover, two neonates were born prematurely due to severe maternal respiratory failure. Outcomes of pregnancies depended on trimesters of pregnancies. Full-term healthy infant (N=1), spontaneous abortion (N=4) and elective termination of pregnancy (N=2) due to social reasons after recovery from SARS were the outcomes of seven pregnant mothers infected with SARS in the first trimester (16). Four preterm labor deliveries with gestational age after 24 weeks were also reported by studying five women (15). Similarly, results acquired from 13 pregnant patients with MERS-CoV showed two preterm labors and two fetal demises. Evidence of vertical transmission was neither detected in SARS nor in MERS (15). However, the limited sample size of pregnant patients infected by COVID-19 found that the behavior of this virus in pregnancy is different. Although the rate of preterm labor and fetal distress was significant, fewer adverse maternal and neonatal outcomes and complications have been reported due to COVID-19 infection in pregnancy (17). Until now, there is no report on the death of pregnant women in articles. There are some reasons for these differences. First of all, the number of cases in these studies is small. Secondly, all women were in their third semester of pregnancy and most of them gave birth less than seven days after the diagnosis of the disease. Hence, this short clinical manifestation-to-delivery time may be too short to affect pregnancies. 

The preferred mode of delivery in cases reported in this review was cesarean section and only five women (6%) delivered vaginally; none of whose neonates was infected with COVID-19. Intrapartum transmission was the main concern for choosing cesarean section. Since there is limited evidence about vertical transmission and vaginal shedding of virus, vaginal delivery in stable patients may be considered. In cases of cesarean section, the choice of anesthesia needs careful consideration (16). Favre et al. suggested that for every individual patient, vaginal delivery even by induction should be considered. Using instrumental delivery also is preferred to cesarean section to avoid unnecessary surgical complications and maternal exhaustion (18).

The main objective of 3 of the 9 original articles included in this review was evaluating the possibility of vertical transmission of COVID-19 in pregnancy. The authors attempted to examine conception products, namely samples of amniotic fluid, cord blood, breast milk, placenta tissue, and vaginal mucus, and assessed RT-PCR results in neonates to answer this critical question. However, neither these samples nor neonates were infected with COVID-19. The most significant point is that all samples were collected during parturition in the operating room. This can ensure that none of the samples were contaminated and also represent the real intrauterine conditions (19). These articles suggested that the possibility of vertical transmission of infection in pregnant women with COVID-19 pneumonia in late pregnancy is trivial. However, these results could be affected by small sample size, mode of delivery and gestational age at the time of infection (20). 

Serious suspicion to the presence of the virus in body secretions led to a study conducted by Cui et al. to explore the possibility of sexual transmission of COVID-19. They analyzed vaginal environment samples such as vaginal discharge and cervical or vaginal residue exfoliated cells as well as anal swab samples in 35 female patients using RT-PCR to detect SARS-CoV-2 (21). Only one anal swab sample was positive for SARS-CoV-2. All the samples acquired from vaginal surroundings were negative for SARS-CoV-2. They suggested that this could be due to the absence of SARS-CoV-2 receptor (ACE2 expression) in vagina and cervix tissues (21). 

Although most of the neonates included in this study did not have any adverse outcomes, the results of the study by Zhu et al. indicated that perinatal COVID-19 infection can cause adverse neonatal outcomes including preterm labor, thrombocytopenia due to abnormal liver function, fetal distress and respiratory distress (8). These results are consistent with neonatal consequences in SARS and MERS infections (15). 

***Implications of COVID-19 for pregnant women***

During this extremely delicate time of a rapidly evolving outbreak that has imposed a tremendous threat on public health, more attention should be given to the unique needs of pregnant women (15). Further investigation and isolation should be considered for pregnant women with suspected COVID-19. In confirmed cases, prompt admission of mothers in a negative pressure isolation unit is crucial (16). 

All the medical staff responsible for taking care of COVID-19 patients should utilize personal protective equipment, namely N95 masks, gloves, gown, and goggles. Standard protocol of the management of COVID-19 in pregnancy incorporates early isolation, controlling infection, administrating oxygen, detecting other viral infections, applying early mechanical ventilation in patients with progressive respiratory failure, and administrating antibiotics in cases of risk of bacterial infection. Monitoring of fetus and uterine contraction should be considered. Any planning on delivery of the patients and their conditions should be made through multi-specialty consultations (15).

Ensuring sufficient rest, fluid and electrolytes balance and nutritional support is important. Oxygen saturation and vital signs should be carefully monitored. Considering the level of hypoxemia, supplemental oxygen (60%-100% concentration at a rate of 40 L/min) through high-flow nasal cannula should be administered. In critical cases, mechanical ventilation or even ECMO may be required for ensuring oxygenation (16). 

In critical cases, pregnancy can put the mother and fetus in danger. Therefore, in these cases, early termination of pregnancy is necessary even in premature pregnancies. This situation needs careful consultation with the patient, her family, and an ethical board. It is also essential that pregnant women avoid unnecessary travel, public transport, crowds, and close contact with patients. Above all, maintaining personal and social hygiene is vital (16).

**Table 3:** Summary of clinical, obstetric and neonatal characteristics of pregnant patients and their neonates

**Author**[Ref] **Number of samples**	**Chen et al.** ** [** 7 **] ** 9 pregnant women	**Liu et al.** ** [** 10 **]** 13 pregnant women	**Zhu et al. ** **[** 8 **]** 10 neonates	**Liu et al. [** 12 **]** 3 pregnant women	**Chen** **et al. [**11**]** 17 pregnant women	**Fan et al. [** 2 **]** 2 pregnant women	**Wang [** 3 **] ** 1 pregnant woman	**Li et al. [** 9 **]** 34 pregnant women (16 confirmed)	**Song et al. [** 13 **]** 1pregnant woman
**Clinical characteristics**									
Age (y)	26–40	22 - 36	25-35	30 -34	29.1 (AV*)	29, 34	28	26-37	30
Exposure to Infection, N (%)	9 (100%)	12 (92%)	All (100%)	3 (100%)	3 (18%)	2(100%)	1 (100%)	NA	1 (100%)
Common Symptoms at onset (N)	Fever (7) Cough (4)	Fever (10) Fatigue (10) Dyspnea (3)	Fever (8) Cough (4)	Fever(2) Cough(2)	Fever (4) Cough (4) Chest distress (2)	Fever (2)	Fever	Fever (5) Cough (1)	Fever, Cough, Myalgia
Severe pneumonia/ mechanical ventilation	None	1(7%)	None	None	None	None	1(100%)	None	None
Maternal Death	None	None	None	None	None	None	None	None	None
Diagnosis	qRT-PCR*	qRT-PCR	qRT-PCR	qRT-PCR	qRT-PCR	qRT-PCR	qRT-PCR	qRT-PCR	qRT-PCR
Main reported Laboratory Findings (N)	Lymphopenia (5) Elevated CRP* (6) Increased ALT&AST*(3)	NA	NA	Decreased Albumin (all) Thrombocytopenia (1)	Lymphopenia (5) Elevated CRP (7)	Lymphopenia (1)	Lymphopenia Decreased Albumin Elevated CRP	Lymphopenia (7) Elevated CRP(16)	NA
Abnormal Radiologic findings (GGO)	YES	NA	YES	YES	YES	YES	YES	YES	YES
Treatment	Oxygen therapy(All) Antiviral therapy (6) Antibiotic therapy(All)	NA	NA	Oxygen therapy(All) Antiviral therapy (All) Antibiotic therapy(n=2)	NA	Antiviral therapy (All) Antibiotic therapy (All)	Oxygen therapy Antiviral therapy Antibiotic therapy	Antibiotics(All) Antiviral therapy (4)	NA
**Prenatal characteristics**									
Gestational Age	36 w- 39w,4d	< 28w (N=2) 3^rd^ Trimester (N= 11)	31- 39 w	38w,4d- 40w	<37 w (N=3) ≥37 w (N=14)	36 w5d & 37w	30w	C^#^: 38±0.2W S^#^:38±2.8W	36w,3d
Prenatal comorbidities before the infection, (N)	Gestational HTN*(1), Preeclampsia (1), Inﬂuenza(1)	None	Vaginal bleeding (1)	Hypothyroid (1) GDM* (1)	Gestational HTN(1), GDM* (2) Anemia (5)	Vaginal bleeding (1)	None	HTN, PCO*, HBS* (3) GDM* (3) Hypothyroidism (2), Preeclampsia (1) Sinus tachycardia(1)	None
Complications, (N)	Fetal distress (2) PROM*( 2) Preterm labor (4)	Fetal distress (3) Stillbirth(1)	Fetal distress (6) PROM(3) abnormal cord(2) previa(1) abnormal AF(2) MODS*(1)	Fetal distress (1)	NA	None	None	Fetal distress (3) PROM (1) Placental abruption (1)	None
Mode of delivery(N)	All C/S*	All C/S	C/S(7), NVD (2)	C/S(2), NVD (1)	All C/S (17)	C/S(2)	C/S	C: C/S(14) S: C/S(16)	C/S
Indication of C/S (N)	COVID 19/ Fetal distress(2) pre-eclampsia (1) PROM(2) History of C-section(1) History of stillbirth(1)	COVID 19/ Fetal distress(3) Stillbirth (1) PROM(1)	COVID 19/ Fetal distress (6) Abnormal AF (2), Abnormal Cord (2), Previa (1) PROM(3)	COVID 19	COVID 19	COVID 19/ Persistent fever (1)	COVID 19/ Mother’s admission to ICU	COVID 19	COVID 19/ History of C-section
Onset to delivery (Days)	1-7 D (before delivery)	NA	1-6 D before (4)- Day of delivery (2) 1-3 D after delivery (3)	1-15 D before delivery	NA	7-11 D before delivery	6 D before delivery	2 D before (1) Day of delivery(1) Missing data of others	8 D before delivery
Preterm Labor, N (%)	4 (44%) (>36w)	6, (46%) 32- 36 w	6 (46%)	None	3(18%)	1(36w5d)	1(100%)	C: 4 (23.5%) S: 4 (21.1%)	1(100%)
Assessment of Vertical Transmission	Breast milk AF, Umbilical cord blood,	NA	NA	Placenta tissue, vaginal mucus, Breast milk, Umbilical cord blood, Serum, Urine	NA	Maternal serum, Vaginal swab, Breast milk, Umbilical cord blood, Placenta tissue, AF	AF, Placenta,Umbilical cord blood, Gastric juice, Stool samples	NA	Amniotic fluid, Umbilical cord blood, Breast milk,
Postpartum complication, N (%)	Postpartum fever 6 (67%)	NA	None	None	E: Hypotension, 12 (86%)	None	None	Postpartum Fever, C: 8 (50.0%) S: 6 (33.3%)	None
**Neonates**									
Fetal weight(Gram)	1880- 3820	NA	1520- 3800	3250- 3670	E*: 3,280 (330) G*: 2,780 (180)	2,890 &3400	1830	C:3078.2 ± 565.0 S: 3188.4 ± 520.5	3630
Abnormal Fetal weight	LBW(2)	-	2(SGA) 1(LGA)	None	None	None	LBW	LBW C: 3 (17.6%) S: 2 (10.5%)	None
Apgar score, 1-5 min	8–9 & 9–10	10 & NA	7–10 & 8–10	8&9	7- 9& 9-10	9&10	9-10	9.6 ± 0.5- 10.0	8-9
Vertical transmission	None	None	None	None	None	None	None	None	None
Special pediatric complication (N)	None	NA One died	Shortness ofbreath (6) Fever (2), Tachycardia (1), Gastrointestinal symptoms (4) Death (1)	Chorioamnionitis (1) Meconium Stained Amniotic Fluid (1)	None	Mild pneumonia (1) Lymphopenia (2) Low-grade fever (1) Abdominal distension (1)	NA	None	None
Neonate’s SARS-CoV-2	All negative	All negative	All negative	All negative	All negative	All negative	All negative	All negative	All negative

**Abbreviation:** SARS-CoV-2=severe acute respiratory syndrome coronavirus 2; MV = Mechanical Ventilation PROM=premature rupture of membrane; MODS=multiple organ dysfunction syndrome; C-section=caesarean section, E: Epidural Anesthesia, G: General Anesthesia, AV: average; qRT-PCR: quantitative real-time reverse transcription-polymerase chain reaction; CRP: C-Reactive Protein; ALT: Amino alanine transferase; AST: Aspartate amino transferase; HTN: Hypertension; PCO: Polycystic ovary syndrome; HBS: Hepatitis B; MODS: Multiple organ dysfunction syndrome; C/S: Cesarean section # confirmed& suspected cases

## Conclusion:

In conclusion, available data revealed that clinical manifestations of pregnant women in late pregnancy are similar to those of non-pregnant adults. There is also no evidence that pregnant women are at higher risk of developing COVID-19 infection or are susceptible to severe pneumonia. Considering the negative results of SARS-COV-2 in samples of umbilical cord blood, amniotic fluid, breast milk, vaginal mucus, placenta tissue, and neonatal throat swab, there is also no evidence of intrauterine transmission of COVID-19 infection in the third trimester of pregnancies. It appears that the risk of fetal distress, preterm delivery and premature rupture of membranes rises when COVID-19 onsets in the third trimester of pregnancy. Since there are no reports of pregnant patients in the first or second trimesters of pregnancy, the risk of stillbirth and abortion as a result of COVID-19 is obscure and needs to be clarified. Given the impact of similar infections (SARS and MERS) on outcomes of pregnancies in the first and second trimesters, further studies are needed to evaluate the long-term effects of COVID-19 on pregnancy and neonate outcomes. 

Overall, due to the lack of information on COVID-19 pneumonia in pregnancy, all suspected pregnant women should be systematically screened, monitored and followed up. Another issue is whether natural vaginal delivery increases the likelihood of vertical transmission of the infection and if so, the possible mechanisms need to be clarified. Further investigations and follow-up studies of pregnant mothers infected by COVID-19 are warranted.

## Limitation:

Studies published in Chinese were not included in this study due to language strain. So, the language of studies had restricted the search and this study is limited by the small sample size. 
